# Dietary supplementation and doping-related factors in high-level sailing

**DOI:** 10.1186/1550-2783-9-51

**Published:** 2012-12-07

**Authors:** Jelena Rodek, Damir Sekulic, Miran Kondric

**Affiliations:** 1 Faculty of Kinesiology, University of Split, Teslina 6, Split, 21000, Croatia; 2 NIHON doo, Spinutska 65, Split, 21000, Croatia; 3 Faculty of Sport, University of Ljubljana, Gortanova 22, Ljubljana, 10000, Slovenia

**Keywords:** Nutritional supplementation, Substances, Testing design, Athlete, Coach

## Abstract

**Background:**

Although dietary supplements (DSs) in sports are considered a natural need resulting from athletes’ increased physical demands, and although they are often consumed by athletes, data on DS usage in Olympic sailing are scarce. The aim of this study was to study the use of and attitudes towards DSs and doping problems in high-level competitive sailing.

**Methods:**

The sample consisted of 44 high-level sailing athletes (5 of whom were female; total mean age 24.13 ± 6.67 years) and 34 coaches (1 of whom was female; total mean age 37.01 ± 11.70). An extensive, self-administered questionnaire of substance use was used, and the subjects were asked about sociodemographic data, sport-related factors, DS-related factors (i.e., usage of and knowledge about DSs, sources of information), and doping-related factors. The Kruskal-Wallis ANOVA was used to determine the differences in group characteristics, and Spearman’s rank order correlation and a logistic regression analysis were used to define the relationships between the studied variables.

**Results:**

DS usage is relatively high. More than 77% of athletes consume DSs, and 38% do so on a regular basis (daily). The athletes place a high degree of trust in their coaches and/or physicians regarding DSs and doping. The most important reason for not consuming DSs is the opinion that DSs are useless and a lack of knowledge about DSs. The likelihood of doping is low, and one-third of the subjects believe that doping occurs in sailing (no significant differences between athletes and coaches). The logistic regression found crew number (i.e., single vs. double crew) to be the single significant predictor of DS usage, with a higher probability of DS consumption among single crews.

**Conclusion:**

Because of the high consumption of DSs future investigations should focus on real nutritional needs in sailing sport. Also, since athletes reported that their coaches are the primary source of information about nutrition and DSs, further studies are necessary to determine the knowledge about nutrition, DSs and doping problems among athletes and their support teams (i.e., coaches, physicians, and strength and conditioning specialists).

## Background

Olympic sailing classes were first used in sailing (also known as yachting) during the 1896 Olympic Summer Games. Since then, 46 different classes have been used. As of this writing, 8 Olympic classes are currently used. Apart from tactical and strategic factors, performance in Olympic sailing relates directly to the sailors’ ability to overcome the external forces imposed on the boat. For obvious reasons (i.e., competition on the open seas), studies have examined sailing conditions, and most of them examined the physiological background of athletes involved in Laser sailing, the most popular Olympic class [1-13]. In short, the energy demand is mainly satisfied by aerobic metabolism, as indicated by reduced levels of oxygen uptake (approximately 35% VO2max) and high heart rates (approximately 75% HRmax). However, the overall psychophysiological demands of Olympic sailing are most specifically related to sailing competitions and the consequent training regime. Official competitions consist of 8 to 14 races, each with a target time of 60 to 80 minutes, over a 6-day period. During the competition, the athletes often spend several hours (often 5 to 7 hours) on the open sea with a limited supply of food and water while being exposed to different climate and weather conditions. Nutrition and hydration are therefore recognized as very important factors in sailing [13-16], but to the best of our knowledge, the problem of dietary supplementation among a representative sample of sailing athletes has not been sufficiently addressed.

Nutrition cannot replace an athlete’s genetic potential, training regime or overall psychosocial preparation, but the most favorable nutritional strategies have been studied and have often proved beneficial. In short, optimal nutrition can reduce fatigue and injuries, promote recovery from injuries [17,18], optimize the human body’s energy stores, and directly influence athletes’ health status [19,20]. Athletes and their teams strive for the best and most convenient nutritional practices to suit the individual needs of each athlete. In doing so, dietary supplements (DSs), i.e., nutritional ergogenic aids, are valuable supports for regular nutrition. In a broader view, DSs are considered “ergogenic aids” because they have the potential to improve training adaptations and enhance exercise performance [21]. Consequently, DS usage among athletes, the rate of which rarely falls below 50% and sometimes exceeds 90%, is not surprising [22-26].

In the most common description, doping is defined as the occurrence of one or more anti-doping code violations, mostly observable by the presence of a prohibited substance or its metabolites or markers in an athlete’s specimens [27]. The practice of doping is often related to serious health problems [28,29] and claimed as potential causes of death cases in sports [30,31]. Although DSs should be considered a logical and natural consequence of athletes’ increased physical demands [32,33], doping is deemed unethical for performance enhancement [34]. However, the sports community is often concerned about DSs being contaminated with doping substances. Briefly, doping agents (i.e., substances directly prohibited by the World Anti-Doping Code) have been traced in some DSs [35,36]. Such incidences understandably raise concerns about DSs in general.

The number and variety of the athletes’ support team differ considerably from sport to sport, mostly due to financial, organizational, and other factors. Nonetheless, the majority of athletes are most closely connected to their coaches, and it is not surprising that coaches are the most important link between athletes and DS use [37,38].

Because we have found no study that investigated DS in sailing athletes, the first aim of this study was to examine DS consumption and attitudes toward DSs among high-level Olympic sailing athletes and their coaches (the Croatian National Olympic team for the 2010/11 season). Because some previous studies recognized certain relationships between nutritional supplementation and doping factors (i.e., they noted nutritional supplementation as a certain gateway to doping) [39], we investigated some specific doping-related factors and the associations between DSs and doping-related factors in sailing.

## Methods

### Participants

We studied 78 subjects, of whom 44 were athletes (39 males and 5 females; mean age 24.13 ± 6.67 years) and 34 were coaches (33 males and 1 female; 37.01 ± 11.70 years). All were members of the Croatian National Sailing Team. Thirty-one athletes sailed in Olympic sailing classes, while 13 sailed in the intermediate sailing classes (i.e., sailing classes that are preliminary to the physically and technically more demanding Olympic classes). At the time of the study, 28 athletes sailed single-crew, while 16 sailed in double-crew boats. All of the subjects were directly under the patronage of the Croatian Sailing Association and the Croatian Olympic Committee as potential Olympic candidates or future Olympic hopefuls, and more than two-thirds of the athletes and 45% of the coaches achieved International competitive results. The IRB approved the investigation, and all participants consented prior to participation in the study.

### Instruments

The testing was undertaken using the Questionnaire of Substance Use (QSU), an instrument that was previously developed and validated with regard to reliability (89 - 93% of subjects responded equivalently within the test-retest design), while the validity was evidenced by an appropriate level of discriminative validity for different groups of subjects [40-43]. The basic QSU includes questions about attitudes toward DSs, doping factors, sociodemographics, and sport-specific factors. The sport-specific factors were modified specifically for sailing as a sport (see Results for more details). The sociodemographic data included age, sex, and educational level.

Sports-related factors (sport-factors) included sports experience (in terms of years involved in sailing), crew number (one or two), current sailing class (Olympic or non-Olympic), and sports achievement (sports results achieved on a 6-point scale from “local competition” to “medal won at European/World championship in Olympic classes”).

DSs and doping factors were studied through questions about the subject’s self determined knowledge about DSs and doping (two separate questions, self-assessed on a five-point scale ranging from “I have no knowledge at all” to “Excellent”), the athlete’s opinion about doping practices in sailing (4-point scale from “I do not think doping is used” to “Doping is often used”), potential doping habits (4-point scale from “I do not intend to use doping” to “I’ll use it if assured it will help me”), trust in coaches regarding doping and trust in physicians regarding doping (both “Yes-No” questions), the number of times the participant has undergone doping testing (four-point scale from “Never” to “More than five times”), and personal opinion regarding penalties for doping offenses (five point scale from “Doping should be allowed” to “Lifelong suspension”). DS usage was assessed using the response to one main question (possible responses were “Yes”, “From time to time”, and “No”) and separate responses for the consumption of vitamins and minerals, carbohydrates, proteins, isotonics, recovery supplements, energy bars, and other DSs. For all of the DSs, we offered four-point scales (“No”, “Sporadically”, “Often”, “Regularly”). In addition, we asked the athletes who their primary source of information was about DSs (possible answers included coach, physician, friend, and self), and for those who did not consume and/or only sporadically consumed DSs, the reason why they did not use DSs, if applicable (the answer options were “I don’t think it will be useful; I have a proper diet”; “I don’t have sufficient knowledge to use DSs”, “The price is too high”, “I don’t think DSs are healthy”).

Statistics: Counts (frequencies) and proportions were calculated for all of the data. Because of the measurement levels present in the data, a nonparametric Kruskal-Wallis ANOVA test was applied to establish differences between (a) the athletes competing in the Olympic classes and those competing in the non-Olympic classes, (b) single- and double-crew athletes, and (c) athletes and coaches for each of the ordinal variables. Analysis of variance (ANOVA) was used to determine differences in parametric variables (age, sport experience) between groups. Spearman’s rank-order correlation was calculated for sport factors, sociodemographic variables, DSs and doping factors (only for ordinal variables). Separate correlation analyses were performed for coaches and athletes. A logistic regression was performed to determine the independent impact of the sociodemographic factors (age, education) and sport factors (crew number, sailing class, competitive achievement, sport experience) on DS usage. A multiple model was built using all six variables, and the criterion variable (DS usage) was dichotomous (DS nonusers vs. DS users). More precisely, for the purpose of the logistic regression calculation, the athletes who reported “Yes” and “From time to time” for their DS usage were grouped as “DS users”; otherwise, they were categorized as “DS nonusers”.

A statistical significance level of 95% (p < 0.05) was applied. Statistical analyses were performed using Statistica Version 10 (Statsoft, Tulsa, OK, USA).

## Results

The athletes and coaches judge their personal knowledge about nutrition and DSs as average in most cases. More than 77% of the athletes consume some type of DS, and 38% do so on a regular basis. Coaches are well aware about DS practice of the athletes. Although the data are not presented separately in the tables, all five of the female athletes use DSs regularly. More than half of the athletes rely on their coaches’ and/or physicians’ opinions about DS and doping issues, but less than one-fourth of the athletes list their coach and/or physician as their primary source of information on DSs and doping, and almost 50% of the athletes and coaches state that the majority of their knowledge about these issues comes from self-education (Table 1).

**Table 1:** Dietary supplement - nutrition factors and Kruskal-Wallis differences between studied groups

	**A**	**C**	**KW**	**O**	**NO**	**KW**	**C1**	**C2**	**KW**
	**f(%)**	**f(%)**	**(p)**	**f(%)**	**f(%)**	**(p)**	**f(%)**	**f(%)**	**(p)**
**Self-determined knowledge on nutrition and dietary supplements**									
I have no knowledge (1)	2(4.5)	0(0.0)	0.12 (0.73)	0(0.0)	2(15.4)	0.5 (0.48)	2(6.9)	0(0.0)	0.15 (0.69)
Poor (2)	9(20.5)	11(32.4)		7(22.6)	2(15.4)		6(20.7)	3(20.0)	
Average (3)	22(50.0)	15(44.1)		16(51.6)	6(46.2)		12(41.4)	10(66.7)	
Good (4)	10(22.7)	6(17.6)		7(22.6)	3(23.1)		8(27.6)	2(13.3)	
Excellent (5)	1(2.3)	2(5.9)		1(3.2)	0(0.0)		1(3.4)	0(0.0)	
Consumption of the DS*									
No (1)	10(22.7)	8(23.5)	1.51 (0.22)	8(25.8)	2(15.4)	1.63 (0.20)	9(31.0)	1(6.7)	0.9 (0.34)
Yes. but not regularly (2)	17(38.6)	6(17.6)		13(41.9)	4(30.8)		9(31.0)	8(53.3)	
Yes. regularly (3)	17(38.6)	20(58.8)		10(32.3)	7(53.8)		11(37.9)	6(40.0)	
**Trust in coaches regarding DS**									
Yes	26(59.1)			19(61.3)	4(30.8)		15(51.7)	11(73.3)	
No	18(40.9)			12(38.7)	9(69.2)		14(48.3)	4(26.7)	
**Trust in physicians regarding DS**									
Yes	24(54.5)			19(61.3)	5(38.5)		15(51.7)	9(60.0)	
No	20(45.5)			12(38.7)	8(61.5)		14(48.3)	6(40.0)	
**Primary source of information on DS**									
I have no knowledge on this problem	6(13.6)	7(20.6)		2(6.5)	4(30.8)		5(17.2)	1(6.7)	
Coach	10(22.7)	8(23.5)		10(32.3)	0(0.0)		5(17.2)	5(33.3)	
Formal education (school. professional seminars. etc.)	7(15.9)	4(11.8)		2(6.5)	5(38.5)		5(17.2)	2(13.3)	
Self-education (Internet. literature. booklets. etc.)	21(47.7)	15(44.1)		17(54.8)	4(30.8)		14(48.3)	7(46.7)	

*LEGEND*: *A* – athletes; *C* – coaches; *O* – Olympic class athletes; *NO* – Non-Olympic class athletes; *C1* – single crew; *C2* – double crew; frequencies – *f*, percentage - %; *KW* - Kruskall-Wallis test; p – statistical significance for df = 1; number in parentheses presents ordinal values for each ordinal variable; * coaches were asked about DS usage of their athletes.

The self-determined knowledge regarding doping issues tends to be below average, with no significant differences between athletes and coaches. Athletes and coaches share opinions about the occurrence of doping in sailing, and one out of three believe that doping occurs to some extent. Opinions about penalties for doping offences tend to favor rigid penalties, including lifetime suspension from competition. The likelihood of doping is low among the study respondents, and only one athlete declare that he/she was likely to try doping in the future. Sixty percent of athletes recognized doping as an issue of fairness and not primarily as a health-threatening behavior, and there is no significant difference between athletes and coaches in any of the studied doping factors. The Olympic crews were more frequently tested for doping and report a lower likelihood of doping than their non-Olympic peers (Table 2).

**Table 2:** Doping factors and Kruskal-Wallis differences between studied groups

	**A**	**C**	**KW**	**O**	**NO**	**KW**	**C1**	**C2**	
	**f(%)**	**f(%)**	**(p)**	**f(%)**	**f(%)**	**(p)**	**f(%)**	**f(%)**	
**Trust in coaches regarding doping**									
Yes	21(47.7)			16(51.6)	5(38.5)		12(41.4)	6(40.0)	
No	23(52.3)			15(48.4)	8(61.5)		17(58.6)	9(60.0)	
**Self determined knowledge on doping**									
I have no knowledge (1)	4(9.1)	1(2.9)	0.07 (0.8)	2(6.5)	0(0.0)	3.7(0.06)	3(10.3)	1(6.7)	0.3 (0.59)
Poor (2)	16(36.4)	13(38.2)		10(32.3)	2(15.4)		11(37.9)	5(33.3)	
Average (3)	14(31.8)	14(41.2)		9(29.0)	6(46.2)		9(31.0)	5(33.3)	
Good (4)	9(20.5)	5(14.7)		9(29.0)	5(38.5)		5(17.2)	4(26.7)	
Excellent (5)	1(2.3)	1(2.9)		1(3.2)	0(0.0)		1(3.4)	0(0.0)	
**Trust in physicians regarding doping**									
Yes	30(68.2)			23(74.2)	7(53.8)		17(58.6)	9(60.0)	
No	14(31.8)			8(25.8)	6(46.2)		12(41.4)	6(40.0)	
**Testing on doping**									
Never (1)	24(54.5)			14(45.2)	10(76.9)	4.50 (0.03)	19(65.5)	5(33.3)	4.39 (0.04)
Once or twice (2)	8(18.2)			6(19.4)	2(15.4)		5(17.2)	3(20.0)	
2-5 times (3)	6(13.6)			5(16.1)	1(7.7)		2(6.9)	4(26.7)	
More than 5 times (4)	6(13.6)			6(19.4)	0(0.0)		3(10.3)	3(20.0)	
**Doping in sailing**									
I don’t think that it is used (1)	11(25.0)	9(26.5)	0.13 (0.72)	7(22.6)	4(30.8)	0.43	6(20.7)	5(33.3)	0.72 (0.39)
Don’t know - not familiar (2)	18(40.9)	15(44.1)		13(41.9)	5(38.5)	(0.51)	16(55.2)	2(13.3)	
It is used but rarely (3)	12(27.3)	8(23.5)		8(25.8)	4(30.8)		6(20.7)	6(40.0)	
Doping is often (4)	3(6.8)	2(5.9)		3(9.7)	0(0.0)		1(3.4)	2(13.3)	
**Personal opinion about penalties for doping offenders**									
Lifelong suspension (1)	8(18.2)	5(14.7)	0.3 (0.58)	5(16.1)	3(23.1)	0.39 (0.85)	8(27.6)	0(0.0)	0.18 (0.67)
First time milder punishment. second time - lifelong suspension (2)	17(38.6)	18(52.9)		14(45.2)	3(23.1)		8(27.6)	9(60.0)	
Suspension for couple of seasons (3)	13(29.5)	8(23.5)		10(32.3)	3(23.1)		8(27.6)	5(33.3)	
Financial punishment (4)	5(11.4)	1(2.9)		2(6.5)	3(23.1)		4(13.8)	1(6.7)	
Doping should be allowed (5)	1(2.3)	2(5.9)		0(0.0)	1(7.7)		1(3.4)	0(0.0)	
**Potential doping habits**									
If assured it will help me no matter to health hazard (1)	0(0.0)			0(0.0)	0(0.0)	9.07 (0.01)	(0.0)	0(0.0)	0.23 (0.63)
I will use it if it will help me with no health hazard (2)	1(2.3)			0(0.0)	1(7.7)		(0.0)	1(6.7)	
Not sure about it (3)	7(15.9)			2(6.5)	5(38.5)		6(20.7)	1(6.7)	
I do not intend to use doping (4)	36(81.8)			29(93.5)	7(53.8)		23(79.3)	13(86.7)	
**The main problem of doping**									
Doping is mainly health-threatening behavior	17(38.6)	17(50.0)		10(32.3)	7(53.8)		13(44.8)	4(26.7)	
Doping is mainly against fair-play	26(59.1)	17(50.0)		21(67.7)	5(38.5)		15(51.7)	11(73.3)	
Doping should be allowed	1(2.3)	0(0.0)		0(0.0)	1(7.7)		1(3.4)	0(0.0)	

*LEGEND*: *A* – athletes; *C* – coaches; *O* – Olympic class athletes; *NO* – Non-Olympic class athletes; *C1* – single crew; *C2* – double crew; frequencies – *f*, percentage - %; *KW* - Kruskall-Wallis test; p – statistical significance for df = 1; number in parentheses presents ordinal values for each ordinal variable.

Vitamins and minerals are the most frequently used dietary supplements, followed by proteins (amino acids), isotonics and energy bars (Table 3).

**Table 3:** Dietary supplement consumption in studied groups

	**A**	**O**	**NO**	**C1**	**C2**
	**f(%)**	**f(%)**	**f(%)**	**f(%)**	**f(%)**
**Vitamins and minerals**					
No	22(50.0)	13(41.9)	9(69.2)	14(48.3)	7(46.7)
Rarely	4(9.1)	1(3.2)	3(23.1)	2(6.9)	2(13.3)
Occasionally	12(27.3)	11(35.5)	1(7.7)	9(31.0)	3(20.0)
Often	6(13.6)	6(19.4)	0(0.0)	3(10.3)	3(20.0)
**Specific vitamins**					
C vitamin (rarely)	10(22.7)				
C vitamin (occasionally)	3(6.8)				
C vitamin (often)	7(15.9)				
E vitamin (occasionally)	2(4.5)				
**Specific minerals**					
Magnesium (rarely and occasionally)	20(45.5)				
Iron (occasionally and often)	6(13.6)				
Calcium (rarely and occasionally)	6(13.6)				
**Carbohydrates**					
No	29(65.9)	20(64.5)	9(69.2)	18(62.1)	11(73.3)
Rarely (sporadically)	7(15.9)	4(12.9)	(0.0)	3(10.3)	4(26.7)
Occasionally	4(9.1)	4(12.9)	3(23.1)	4(13.8)	0(0.0)
Often	4(9.1)	3(9.7)	1(7.7)	4(13.8)	0(0.0)
**Proteins/Amino acids**					
No	26(59.1)	17(54.8)	9(69.2)	16(55.2)	10(66.7)
Rarely (sporadically)	3(6.8)	1(3.2)	2(15.4)	2(6.9)	1(6.7)
Occasionally	12(27.3)	10(32.3)	2(15.4)	8(27.6)	4(26.7)
Often	3(6.8)	3(9.7)	0(0.0)	3(10.3)	0(0.0)
**Isotonic drinks**					
No	25(56.8)	15(48.4)	10(76.9)	16(55.2)	9(60.0)
Rarely (sporadically)	4(9.1)	2(6.5)	2(15.4)	4(13.8)	0(0.0)
Occasionally	12(27.3)	11(35.5)	1(7.7)	7(24.1)	5(33.3)
Often	3(6.8)	3(9.7)	0(0.0)	2(6.9)	1(6.7)
**Combined recovery supplements**					
No	25(56.8)	15(48.4)	10(76.9)	20(69.0)	5(33.3)
Rarely (sporadically)	10(22.7)	8(25.8)	0(0.0)	3(10.3)	7(46.7)
Occasionally	8(18.2)	8(25.8)	2(15.4)	5(17.2)	3(20.0)
Often	1(2.3)	0(0.0)	1(7.7)	1(3.4)	0(0.0)
**Energy bars**					
No	19(43.2)	12(38.7)	7(53.8)	15(51.7)	4(26.7)
Rarely (sporadically)	8(18.2)	6(19.4)	2(15.4)	4(13.8)	4(26.7)
Occasionally	17(38.6)	13(41.9)	4(30.8)	10(34.5)	7(46.7)
Often	0(0.0)	0(0.0)	0(0.0)	0(0.0)	(0.0)
Something else*					
Echinacea	4(9.1)				
Propolis	2(4.5)				
Spirulina	3(6.8)				
L carnitine	1(2.3)				
Other	3(6.8)				

*LEGEND*: *A* – athletes; *O* – Olympic class athletes; *NO* – Non-Olympic class athletes; *C1* – single crew; *C2* – double crew; frequencies – *f*, percentage - %; * percentage is calculated for all athletes.

**Figure 1:**
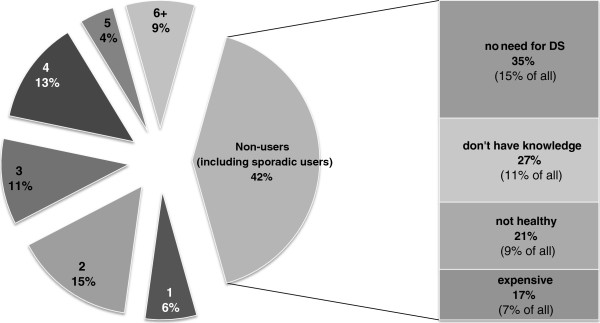
Athletes’ self-reported use of different dietary supplements (for dietary supplement users), and reasons for not using dietary supplements (for non-users and sporadic users).

More than 13% of the athletes use five or more DSs, and the main barriers to DS use vary between athletes (Figure 1).

DS use is less frequent among older athletes and those who achieved higher-level competitive results, while those who achieved greater competitive success were tested more often for doping. The frequency of doping testing is negatively related to DS use. Self-reported knowledge about doping is correlated with self-reported knowledge about nutrition and DSs (Table 4).

**Table 4:** Correlation analysis between ordinal variables for athletes

	**Sport achievement**	**Knowledge on nutrition and DS**	**Knowledge on doping**	**Consumption of the DS**	**Testing on doping**	**Doping in sailing**	**Penalties for doping**
Age	0.41*						
Sport experience	0.48*						
Sport achievement	-						
Knowledge on nutrition and DS	-0.01	-					
Knowledge on doping	0.09	0.58*	-				
Consumption of the DS	-0.32*	-0.19	-0.27	-			
Testing on doping	0.67*	0.25	0.31*	-0.47*	-		
Doping in sailing	0.30	0.04	0.08	-0.15	-0.21	-	
Penalties for doping	0.13	-0.03	0.07	0.10	0.12	-0.21	-
Doping likelihood	-0.04	0.16	0.16	-0.04	0.19	-0.05	-0.18

*LEGEND*: * denotes significant correlation coefficients at p < 0.05.

A logistic regression analysis reveals that “crew number” is the single significant predictor of DS usage among the factors, and this single-variable model is the only significant logistic model built (p < 0.05). The model (Y = -1.042 + 1.841 * X) successfully classified 67% DS users and 32% DS nonusers, indicating that single crews as more inclined to DS usage (OR: 1.4-2.2).

## Discussion

In the following text we will discuss the findings we have judged to be the most important with regard to study aims and topics that have not been previously investigated (i.e., types of DSs consumed, opinions about doping in sailing). Therefore, the discussion will focus on DS use habits in conjunction with DS-related factors and doping likelihood.

Our data revealing that 70% of sailing athletes are DS users support figures of other studies which have reported that the percentage of supplement users ranges from 60% to 93% [22-26,44,45]. Therefore, although the previous studies did not assess DS use the way we did (i.e., previous studies examined DS habits on a nominal “yes-no” scale, while we used a ordinal scale; see the tables for more details), our findings that 38% of athletes used DSs occasionally and an additional 38% used them regularly are among the highest reported prevalence of DS use among athletes. Given the characteristics of sailing and the associated training and competition (see Introduction and following text for details), such a relatively high incidence is expected.

The reasons why vitamins, minerals and isotonic (electrolyte) drinks are consumed in most cases, and why most athletes use them regularly, are related to the characteristics of the sport of sailing. Both competitions and training of sailing often last for more than 5 hours. The athletes are regularly far away from the coast, and they wear sailing suits made of neoprene and latex materials that do not allow regular perspiration. It has already been noted that most of the sailing athletes are in a negative fluid balance after racing (mean loss for males: - 2.1%; for females: - 0.9%) [14]. In addition, Croatia is a Mediterranean country with a temperature ranging from 15 to 30 degrees Celsius (from March through the end of September, when most sailing occurs), and it is clear that adequate rehydration is difficult to achieve without isotonic drinks. Because hot-cold and dry-wet changes are common (i.e., weather conditions can change considerably during a single training session) and frequent travel is required (i.e., when the athletes are not at home), athletes’ diets are unintentionally incomplete, daily multivitamin/mineral supplementation has been recognized as an “insurance policy” for health promotion and disease prevention [46].

Although most vitamin supplements combine several of the most important minerals and microelements, our results showed that mineral consumption is mostly confined to magnesium (Mg) supplementation. The background of such practices will be briefly explained from the perspective of an “insider” in sailing (i.e., one of the authors is directly involved in competitive sailing), and it is mostly related to muscle cramps and problem of constipation. The sport of sailing combines static and dynamic muscular endurance, and leg cramps frequently occur, especially during prolonged competitions (see Introduction for details about the organization of the main competitions in sailing). Mg is considered valuable for the treatment of muscle cramps in general and not only in sports [47-49], and some of the sailing athletes follow such practice. Additionally, Mg (magnesium oxide) is a known medical treatment for functional constipation [50]. Although constipation is generally very rare among athletes in general, it is a known concern among competitive sailors. Most often, the athletes and coaches are responsible for transporting their gear by vehicle, and during travel, constipation is not unusual. This is not surprising because under such circumstances, all five of the main causes of constipation [51] are present: “fiber-deprived food”(i.e., sandwiches), inactivity (i.e., prolonged sitting), lack of liquid (i.e., drinking increases the need to urinate, which is obviously a problem while driving), ignoring the urge to go to the toilet, and stress (because of the upcoming competition). Although we did not study it systematically, our experience is that acute Mg supplementation effectively solves the problem of constipation, and such supplementation is known practice among the sailing athletes who participated in our study.

Our findings of a negative relationship between age and supplement use are in clear disagreement with previous studies, which in most cases noted more frequent DS consumption among older athletes [22,45,52]. The most probable reason for this inconsistency is the age of the subjects. Sailing is a sport where athletes of advanced age can compete at high levels. Therefore, the mean age of our subjects was 24 years, and 20% of the athletes were older than 30 years. Our colleagues [22,45,52] who reported a higher rate of DS usage among older athletes studied younger subjects (from 16.6 to 21.2 years of age) than we did. This most likely explains why we found a numerically low but significant negative relationship between competitive achievement and DS usage. In short, older athletes (i.e., those who consume fewer DSs) are more likely to achieve higher-level competitive results (i.e., they have had more chances to win medals at advanced levels of competition).

Sailing in a single crew is the only factor that significantly predicts more frequent DS use (note that we included all sociodemographic and sport-related factors in logistic regression, as potential predictors of DS use). The background for such a finding will be briefly discussed. Sailing is known to be a “tactical sport”, especially during low wind speed conditions. During high wind speed conditions, the energy demands of sailing increase [6]. For double crews, the boat and the gear are generally larger than for single crews; however, this difference mostly adds to the tactical and technical demands of the sport and not to the physical demands. It can be said that the overall physical demand on each member of the double crews is lower than the physical demand on the athletes who compete in a single crew [53], which results in lower DS consumption among double crews.

The likelihood of doping among Croatian competitive sailors is relatively low and is lower than that reported previously for other athletes from the former Yugoslavia [42,43,54,55]. The reason for such encouraging findings is most likely related to the facts that (I) sailing is a sport that has not been contaminated by doping [56], while (II) sailing athletes we have studied do not believe that doping occurs in sailing. The later is especially important knowing that the belief that doping persists in a particular sport is the most significant risk-factor for future doping behavior [43].

In some recent studies, nutritional supplementation was found to be a potential gateway to doping [39]; however, the findings seem to be sport-specific and most likely culturally specific, as other studies concluded the opposite (i.e., that there is a higher likelihood of potential doping behavior in DS nonusers) [43]. Mostly because of the very low doping likelihood (i.e., only one sailing athlete reported possibly engaging in doping behavior in the future but only if convinced that there would be no health-related consequences), we could not study the problem more specifically and therefore cannot support either of the two opposing findings regarding the influence of current DS practice on the likelihood of doping.

With regard to nutrition, DSs and doping, the athletes’ trust in their coaches is absolutely crucial, mostly because of the possible misinterpretations and misunderstandings related to DSs and doping [57]. Furthermore, nutrition and DSs are long-term investments in the athletes’ development, and the effect of proper dietary habits and DS consumption is difficult to observe in the short term. Studies that investigate the issue of athletes’ trust in their coaches regarding DSs and doping in our territory (former Yugoslavia) are generally disappointing, and trust in coaches regarding DS and doping is rarely reported in more than 40% of studied athletes [42,43]. Therefore, we find it encouraging that “only” 40% of sailing athletes do not trust their coaches regarding DSs and that 50% do not trust them regarding doping. Interestingly, the relatively high percentage of sailors who rely on their coaches’ advice about DSs and nutrition is not related to the number of the athletes who declared their coaches as the primary source of information on nutrition and DS. The finding of 22% sailing athletes who stated that their coaches are the first source of information on these topics is lower than those presented previously for other countries [37,38]. Because an almost equal proportion of coaches and athletes reported “self-education” as the main source of their DS and nutrition knowledge, it is logical to conclude that sailing athletes and coaches essentially learn about these topics together.

The issue of “self-education” in nutrition and DS use deserves special attention. We must stress that although understandable (i.e., until approximately 20 years ago, sports nutrition was not systematically studied and reported as a valuable support to sports achievement, and therefore it was rarely included into formal educational systems), self-education can be particularly dangerous, especially with regard to the dissemination of incorrect information. Like training and/or sports gear, nutrition and DS use are efficient only in so far as they are appropriately chosen (with regard to the athlete’s specific needs) and adequately consumed (with regard to amount, frequency and timing). In addition to the potential lack of efficiency if used incorrectly, it is important to note that the inadequate selection and consumption of DSs and polypharmaceuticals can lead to serious health problems [58]. The main problem is the possible dissemination of incorrect information that is not supported by research and practice. This problem directly relates to the previously stated need for DSs and knowledge of DSs. We believe that the interrelationship between these two factors is an indicator of the appropriateness and, consequently, the potential benefits of DSs.

An important aspect of this investigation was the aim of identifying potential differences in DS use and doping factors between athletes and their coaches. Therefore, the coaches were asked questions similar to those the athletes answered. The idea was to determine (I) whether the coaches are informed about the athletes’ DS use, (II) whether there is a difference between athletes and coaches regarding their opinions about doping in sailing, and (III) whether the opinions of the athletes and coaches regarding potential doping behavior are similar. As far as our study design allows us to determine, it seems that (I) coaches are well informed about their athletes’ DS practices, (II) athletes and coaches share the same opinions about doping in sailing, (III) athletes and coaches have similar attitudes about potential doping behavior, and (IV) there is no significant difference between athletes and coaches with regard to self-reported knowledge regarding doping and nutrition. It seems that the specific characteristics of sailing (e.g., traveling and living together) lead to a very close coach-athlete relationship in which the obligations of one side are the rights of the other, and any type of knowledge is shared between them. Under such conditions, it is difficult to imagine that coaches would not know what DSs their athletes are consuming.

### Study limitations

The limitations of these results and the conclusions drawn from them stem mostly from the self-reported nature of the study data and the fact that we studied relatively small sample from only one country. First, this investigation is based on the subjects’ self reports. The subjects might not have told the truth, especially if they felt uncomfortable. However, we believe that the testing design (see Materials and methods) and experience gained from previous studies decreased this possibility. Second, we must note that this study relies on subjects sampled from only one country; therefore, any generalizations are questionable. However, because Croatia’s excellence in this sport is widely recognized and because we studied all of the subjects we intended to include in the study (the entire National team, a 100% response rate), we believe that although the data presented and discussed in this study are not the final word on the subject, they should be considered a significant contribution to the knowledge in the field. Finally, one of our aims was to compare athletes and coaches’ opinions about and attitudes toward DSs and doping, but we were unable to do so accurately because of the need for an anonymous investigation. In other words, we could not compare each athlete’s responses to those of his/her coach.

## Conclusion

Although the high frequency of DS usage among sailing athletes can be explained by the characteristics of the sport (i.e., athletes being on the open sea for several hours, challenging weather conditions, and long drives), there is a need for further investigation of the exact nutritional needs of those athletes. Such an analysis will not only provide more detailed insight into the real nutritional value and necessity of DSs but also prevent possible misuse and overconsumption of DSs. Additionally, the results clearly highlight the need for a precise analysis of the differences between single and double crew members in real sailing conditions, especially with regard to physiological background and eventual nutrient deficiencies.

In addition to the opinion that DSs are useless, a self-declared “lack of knowledge about DSs” was found to be an important reason for avoiding DSs. Therefore, future studies should seek out precise information about athletes’ knowledge of nutrition, DSs and doping problems in sailing. In doing so, special attention should be paid to supporting team members (coaches, physicians, athletic trainers, strength and conditioning specialists) and their knowledge, as the athletes reported that coaches are the primary source of information about nutrition and DSs. Because our ability to investigate this variable was seriously limited (i.e., due to the problem of anonymity, we could not compare each athlete with his/her coach), future studies should focus on this problem using some specific testing designs which will not influence the anonymity but will more accurate assure comparison between athletes and their coaches (i.e., by testing athletes and coaches anonymously but asking them to use paired codes as identification).

## Competing interests

The authors declare that they have no competing interests.

## Authors’ contributions

JR performed statistical analysis and discussed data. DS designed the testing procedure, collected the data, and discussed the results; MK did preliminary statistical procedures and drafted the manuscript. All authors have read and approved the final version.
